# Reversible Dementia With Hypereosinophilia: An Intricate Connection

**DOI:** 10.7759/cureus.86009

**Published:** 2025-06-14

**Authors:** Ajay Emani, Ramakant Yadav, Roopesh Kirar, Midhun Mohan

**Affiliations:** 1 Neurology, Uttar Pradesh University of Medical Sciences, Saifai, IND

**Keywords:** cortical atrophy, hypereosinophilia syndrome, loss of memory, reversible dementia, t2 hyperintensities

## Abstract

Hypereosinophilic syndrome (HES) is a rare group of disorders characterized by elevated eosinophil levels, leading to tissue infiltration and damage. Neurological complications are observed in over half of the patients. This case study discusses a 45-year-old male patient diagnosed with HES and rapidly progressive dementia. The patient presented with difficulties in routine tasks, attention deficits, memory loss, and frontal headaches lasting one year. His medical history included allergic rhinitis and eczematoid skin lesions. General and neurological examinations revealed deficits in higher mental functions without other abnormalities. Laboratory tests showed significant eosinophilia, and magnetic resonance imaging (MRI) of the brain indicated diffuse cortical atrophy and periventricular hyperintensities. The diagnosis of HES was supported by clinical and laboratory findings. The case highlights the importance of considering HES in patients with unexplained neurological symptoms and eosinophilia to prevent irreversible organ damage. Early recognition and appropriate management are crucial for improving patient outcomes. This study underscores the need for further research to understand HES's pathophysiology and develop targeted therapies.

## Introduction

Hypereosinophilic syndrome (HES) refers to a rare and diverse group of disorders marked by sustained and significant increases in blood eosinophil levels, resulting in the release of inflammatory mediators that cause tissue infiltration and damage [1]. Neurological complications such as ischemic strokes, encephalopathy, peripheral neuropathy, venous sinus thrombosis, and/or dementia occur in over half of the patients [2]. We present a case of HES associated with rapidly progressive dementia. The criteria for HES are one or more of the following: (i) Hypereosinophilia (HE) is characterized by >1.5 x 109 eosinophils on CBC on 2 examinations >1 month apart; (ii) The percentage of eosinophils in the bone marrow section must exceed 20% of all nucleated cells; (iii) The pathologist's assessment that the tissue infiltration by eosinophils is extensive and/or marked deposition of eosinophil granule proteins is found; (iv) Evidence of organ or tissue damage attributable to tissue HE; (v) Exclusion of other disorders or conditions as major reasons for organ damage [1].

## Case presentation

A 45-year-old male patient presented with chief complaints of difficulty in carrying out his routine activities associated with forgetfulness in the form of attention deficits with memory loss last one year. He also had associated headaches in the frontal region for one year. Upon further eliciting history, we were informed that he has suffered from allergic rhinitis with eczematoid skin lesions. There was no significant past or family history.

On general examination, the patient was conscious and cooperative with stable vitals, pulse 90/min, and blood pressure 124/78 millimeters of Hg, without pallor, cyanosis, icterus, or edema. Neurological examination revealed deficits in higher mental functions in the form of Mini Mental State Examination Score of 05/30 with poor frontal assessment battery score of 3/18, losing points in attention, lexical fluency, motor luria, and abstract thinking. The rest of the neurological examination did not show any significant abnormality. Muscle strength and tone were normal. Sensory examination was intact. Deep tendon reflexes were normal. Lymph nodes and the thyroid gland were not palpable. There were no hepato-splenomegaly or palpable masses. There was no other evidence of systemic diseases.

Routine investigations and investigations such as HIV and RPR to rule out other causes of dementia were carried out, but they were negative. Electroencephalogram was also done, which showed no evidence of periodic sharp wave discharges to consider the possibility of prions disease. Table 1 shows the various relevant blood and CSF investigations taken during the hospital course.

**Table 1:** Laboratory fluid investigations

Laboratory investigation	Patient value	Normal value
Hemoglobin	14.9 gm/dl	13-17 gm/dL
Total leukocyte count	19,700/cmm	4000-10,000/cmm
Neutrophils	24.7%	40-80%
Lymphocytes	12.9%	20-40%
Eosinophils	57.8%	2-10%
Monocytes	4%	1-6%
Basophils	1.6%	<2%
Platelets	1.15 lakhs/cmm	1.5-4.1 lakhs/cmm
ESR	18 mm/hour	>20 mm/hour
S. creatinine	0.68 mg/dL	0.67-1.17 mg/dL
Sodium	139 mEq/dL	136-146 mEq/L
CSF protein	23.91 mg/dL	15-45 mg/dL
CSF glucose	103 mg/dL	60-100 mg/dL
CSF cells	<5 cells	<5 cells

**Figure 1:**
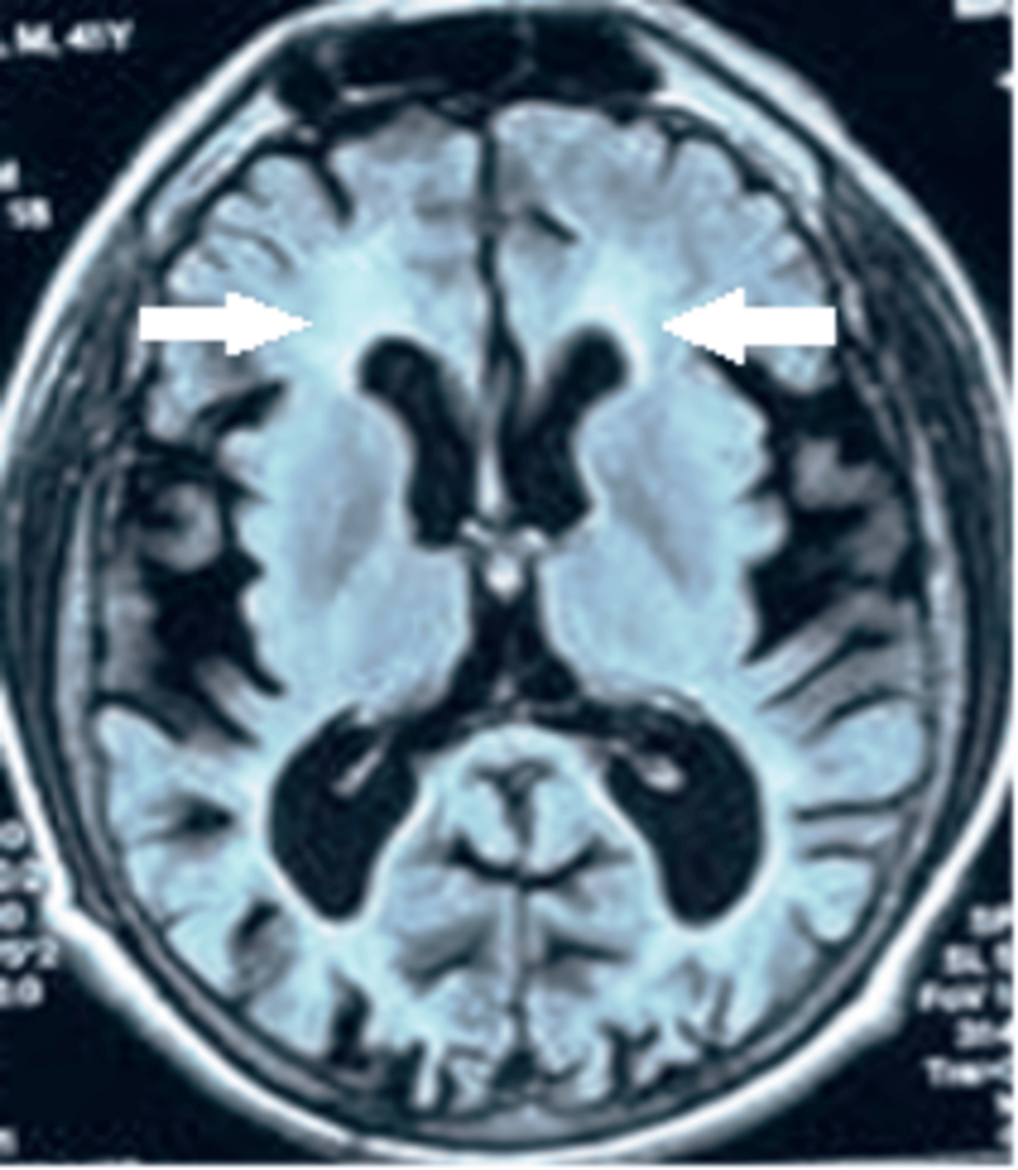
Periventricular T2/FLAIR hyperintensities with diffuse cortical atrophy

Chest X-ray was normal. MRI brain showed the presence of diffuse cortical atrophy with periventricular T2/FLAIR hyperintensities. Thyroid function tests were normal. Neuroimaging in the form of MRI brain showed the presence of diffuse cortical atrophy with periventricular T2/FLAIR hyperintensities (Figure 1).

Based on clinical findings and investigations, HES was considered as a strong possibility for the cause of dementia and hence, the patient was started on pulse therapy of steroids for a period of five days following which the patient improved such that his MMSE was 22-23/30 and he was better oriented to time, place, and person.

## Discussion

HES is characterized by chronically increased peripheral blood eosinophil levels and organ damage related to eosinophilic infiltration [3]. The incidence of HES ranges from 0.16 to 0.36 per 100,000, with a prevalence between 0.36 and 6.3 per 100,000 [4].

Eosinophils, as highly adaptable leukocytes, possess strong pro-inflammatory, pro-thrombotic, and pro-fibrotic characteristics. Their diverse functions can lead to tissue infiltration, ultimately causing end-organ damage and dysfunction [5,6].

Neurological manifestations of HES can involve both the central and peripheral nervous systems. Central nervous system (CNS) involvement may present as encephalopathy or organic psychosyndrome. Peripheral nervous system involvement, which constitutes over 50% of neurological symptoms in HES, can manifest as peripheral neuropathy, mononeuropathy multiplex, autonomic neuropathy, or polymyositis [7].

A direct infiltration of eosinophils, a microvascular permeability alteration [8,9], and toxic effects of eosinophil-derived proteins [10] may account for HES encephalopathy. Neurotoxicity of human eosinophils has been shown experimentally [11]. The lack of eosinophils in the cerebrospinal fluid (CSF) is not inconsistent with an eosinophil-mediated encephalopathy, as CNS damage is likely mediated by circulating eosinophils' toxic products. The only report of CSF eosinophilia was a case in the setting of Giardia infection [12].

In our case, the patient presented with progressive forgetfulness and frontal headache. MRI of the brain demonstrated cortical atrophy with associated periventricular changes. Hematological analysis revealed peripheral eosinophilia. Following the administration of methylprednisolone, the patient exhibited significant clinical improvement such that his MMSE increased up to 22-23/30 and eosinophil was within the normal limit. The patient has been on monthly follow-up for three months although a repeat MRI could not be done due to financial constraints. Since the clinical improvement was so significant, it was not deemed mandatory for the patient.

Consistent with other studies, serum eosinophilia is associated with disruption of the blood-brain barrier, which results in apoptosis of brain cells, leading to dementia, thus explaining the apoptosis. Hence, neither CSF eosinophilia nor biopsy is mandatory to establish the diagnosis; however, they could not be done due to the lack of facilities.

We believe that the recognition of asymptomatic leukoencephalopathy and/or cortical atrophy in HES should prompt a careful follow-up of the patient in order to start an appropriate treatment as soon as encephalopathic symptoms become evident.

## Conclusions

HES can lead to neurological complications due to eosinophilic infiltration and toxicity. Our case highlights the association between HES and cortical atrophy with periventricular changes, emphasizing the importance of early recognition and treatment. The patient's clinical and hematological improvement following methylprednisolone therapy suggests a reversible immune-mediated process. Close monitoring of asymptomatic leukoencephalopathy in HES is crucial for timely intervention to prevent irreversible neurological damage.
